# Supplementary material for: Air Pollution and Preterm Birth: Do Air Pollution Changes over Time Influence Risk in Consecutive Pregnancies among Low-Risk Women?
Source: Int J Environ Res Public Health. 2019 Sep 12;16(18):3365. doi: 10.3390/ijerph16183365 (PMC6765877; doi:10.3390/ijerph16183365)
Supplement: Supplementary file 1 [file ijerph-16-03365-s001.pdf]

Supplemental Table S1. Cutpoints for high, moderate and low exposure levels based on the first observed pregnancy among 50,005 mothers, NICHD Consecutive Pregnancy Study, 2002-2010

	First pregnancy (n=50,005)		
	Low	Moderate	High
Sulfur dioxide (ppb)	0.73-1.57	1.57-2.20	2.20-3.07
Ozone (ppb)	22.49-38.91	38.91-43.70	43.70-48.59
Nitrogen oxides (ppb)	12.65-28.94	28.94-40.35	40.35-67.30
Nitrogen dioxide (ppb)	9.67-15.07	15.07-19.47	19.47-29.74
Carbon monoxide (ppb)	246.77-488.48	488.48-675.08	675.08-1045.47
Particulates <2.5 microns (µ/m3)	5.31-7.50	7.50-9.75	9.75-15.28
Particulates <10 microns (µ/m3)	14.51-20.74	20.74-25.29	25.29-32.71

Supplemental Table S2. Distribution of air pollutants in first and second pregnancy for whole pregnancy exposure and average exposure in first 28 weeks of pregnancy among 50,005 mothers, NICHD Consecutive Pregnancy Study, 2002-2010

	First pregnancy					Second pregnancy				
	min	P25	median	P75	max	min	P25	median	P75	max
Whole pregnancy										
SO_2	0.73	1.57	1.94	2.20	3.07	0.98	1.57	2.00	2.12	3.04
O_3	22.49	38.91	41.59	43.70	48.59	17.83	32.73	37.14	39.80	46.60
NO_X	12.65	28.94	35.07	40.35	67.30	11.28	25.21	30.54	35.66	61.79
NO_2	9.67	15.07	17.42	19.47	29.74	8.11	13.92	16.01	18.36	28.31
CO	246.77	488.48	586.32	675.08	1045.47	221.19	402.60	477.28	554.74	898.98
$PM_{2.5}$	5.31	7.50	8.63	9.75	15.28	5.38	7.72	8.36	9.35	14.99
PM_{10}	14.51	20.74	22.90	25.29	32.71	15.62	20.25	22.37	24.02	32.42
28 weeks gestation										
SO_2	0.73	1.58	1.93	2.21	3.12	0.96	1.62	1.96	2.16	3.12
O_3	20.26	39.39	42.10	43.74	48.62	17.82	32.40	37.43	41.08	47.50
NO_X	11.36	25.19	35.60	45.67	71.73	10.21	21.60	30.78	39.56	71.71
NO_2	8.72	14.72	17.19	20.55	31.19	7.66	13.35	15.97	19.18	31.19
CO	245.86	475.30	584.28	700.86	1077.43	209.11	389.12	481.44	574.65	955.72
$PM_{2.5}$	5.18	7.10	8.39	10.22	15.18	5.42	7.26	8.46	9.39	15.02
PM_{10}	14.61	20.43	22.99	25.73	33.28	15.31	19.88	22.15	24.17	32.65

Supplemental Table S3. Change in air pollution category from first to second pregnancy by preterm birth in first pregnancy among 50,005 mothers, NICHD Consecutive Pregnancy Study, 2002-2010

	Preterm		No preterm	
	n	%	n	%
SO_2				
Stay high	409	10.8	4488	9.7
Stay moderate	1006	26.6	12975	28.1
Stay low	609	16.1	8375	18.1
Increase	746	19.7	7939	17.2
Decrease	1016	26.8	12442	26.9
O_3				
Stay high	352	9.3	3969	8.6
Stay moderate	813	21.5	11200	24.2
Stay low	202	5.3	2288	5.0
Increase	175	4.6	1494	3.2
Decrease	2244	59.3	27268	59.0
NO_X				
Stay high	455	12.0	5349	11.6
Stay moderate	764	20.2	12101	26.2
Stay low	369	9.8	3707	8.0
Increase	672	17.8	5983	12.9
Decrease	1526	40.3	19079	41.3
NO_2				
Stay high	520	13.7	5707	12.4
Stay moderate	881	23.3	12758	27.6
Stay low	404	10.7	5392	11.7
Increase	524	13.8	4658	10.1
Decrease	1457	38.5	17704	38.3
CO				
Stay high	459	12.1	4947	10.7
Stay moderate	852	22.5	12511	27.1
Stay low	361	9.5	3821	8.3
Increase	324	8.6	2136	4.6
Decrease	1790	47.3	22804	49.3
$PM_{2.5}$				
Stay high	292	7.7	3060	6.6
Stay moderate	743	19.6	11159	24.1
Stay low	314	8.3	3648	7.9
Increase	1219	32.2	13886	30.0
Decrease	1218	32.2	14466	31.3
PM_{10}				
Stay high	443	11.7	4490	9.7
Stay moderate	951	25.1	13141	28.4
Stay low	625	16.5	7943	17.2
Increase	684	18.1	7187	15.6
Decrease	1083	28.6	13458	29.1

Note: Categories are based on first pregnancy distribution. High >75th percentile; Moderate 25th-75th percentile; Low <25th percentile. Increasing or decreasing is based on a change in category.

Supplemental Table S4. Adjusted relative risk of preterm birth in second pregnancy by change in average criteria pollutant exposure levels for the first 28 weeks of gestation from first to second pregnancy among 50,005 mothers, NICHD Consecutive Pregnancy Study, 2002-2010

Change in exposure from first to second observed pregnancy	Second Pregnancy Preterm Birth			
	RR	LCL	UCL	P
Stay high vs. stay low				
SO_2	1.15	1.02	1.30	0.028*
O_3	0.94	0.81	1.11	0.48
NO_X	1.06	0.91	1.23	0.45
NO_2	1.16	1.01	1.33	0.040*
CO	1.10	0.95	1.27	0.20
$PM_{2.5}$	1.04	0.88	1.22	0.66
PM_{10}	1.22	1.07	1.38	0.002*
Stay moderate vs. stay low				
SO_2	1.15	1.05	1.27	0.004*
O_3	0.99	0.86	1.13	0.87
NO_X	0.96	0.84	1.09	0.54
NO_2	1.07	0.95	1.21	0.27
CO	1.02	0.90	1.16	0.72
$PM_{2.5}$	1.03	0.90	1.18	0.64
PM_{10}	1.11	1.00	1.23	0.050
Increase vs. stay low				
SO_2	1.15	1.04	1.27	0.007*
O_3	0.99	0.84	1.17	0.93
NO_X	0.98	0.86	1.11	0.73
NO_2	1.00	0.88	1.13	0.96
CO	1.02	0.89	1.18	0.75
$PM_{2.5}$	1.03	0.91	1.18	0.61
PM_{10}	1.11	0.99	1.24	0.06
Decrease vs. stay low				
SO_2	1.18	1.07	1.30	0.001*
O_3	0.98	0.86	1.12	0.81
NO_X	0.96	0.85	1.09	0.54
NO_2	1.05	0.93	1.18	0.44
CO	1.00	0.89	1.13	0.95
$PM_{2.5}$	0.98	0.86	1.11	0.75
PM_{10}	1.11	1.00	1.22	0.043*

* Significance of $p < .05$

Covariates: Preterm birth in first pregnancy; interpregnancy interval; maternal age; race/ethnicity; pre-pregnancy BMI; smoking; alcohol use; parity; insurance status; marital status; asthma history

Supplemental Table S5. Adjusted relative risk of preterm birth in second pregnancy by change in average whole pregnancy criteria pollutant exposure levels from first to second pregnancy among 27,137 nulliparous mothers, NICHD Consecutive Pregnancy Study, 2002-2010

	Second pregnancy preterm birth risk			
	RR	LCL	UCL	P
Stay high vs. stay low				
SO_2	1.22	0.95	1.56	0.12
O_3	1.62	1.21	2.18	0.001*
NO_X	1.41	1.08	1.85	0.012*
NO_2	1.38	1.08	1.76	0.010*
CO	1.20	0.92	1.55	0.18
$PM_{2.5}$	0.97	0.73	1.28	0.81
PM_{10}	1.08	0.84	1.40	0.55
Stay moderate vs. stay low				
SO_2	1.07	0.90	1.26	0.47
O_3	1.25	0.99	1.56	0.06
NO_X	0.97	0.80	1.17	0.75
NO_2	0.96	0.80	1.15	0.63
CO	0.88	0.74	1.06	0.17
$PM_{2.5}$	0.81	0.67	0.98	0.026*
PM_{10}	0.88	0.74	1.05	0.15
Increase vs. stay low				
SO_2	1.34	1.15	1.57	0.0003*
O_3	1.80	1.41	2.31	<.0001*
NO_X	1.13	0.94	1.36	0.19
NO_2	1.38	1.15	1.66	0.001*
CO	1.33	1.09	1.64	0.006*
$PM_{2.5}$	0.91	0.77	1.07	0.25
PM_{10}	1.17	1.00	1.37	0.06
Decrease vs. stay low				
SO_2	1.11	0.91	1.36	0.32
O_3	1.28	1.02	1.61	0.035*
NO_X	1.15	0.94	1.42	0.17
NO_2	1.15	0.95	1.39	0.14
CO	0.97	0.80	1.17	0.74
$PM_{2.5}$	0.95	0.76	1.18	0.64
PM_{10}	0.98	0.80	1.21	0.86

* Significance of $p < .05$

Covariates: Preterm birth in first pregnancy; interpregnancy interval; maternal age; race/ethnicity; pre-pregnancy BMI; smoking; alcohol use; parity; insurance status; marital status; asthma history

Supplemental Table S6. Adjusted relative risk of preterm birth in second pregnancy by change in average whole pregnancy criteria pollutant exposure levels from first to second pregnancy by prior preterm birth status among 27,137 nulliparous mothers, NICHD Consecutive Pregnancy Study, 2002-2010

	Preterm birth in first pregnancy				No preterm birth in first pregnancy				Interaction P
	RR	LCL	UCL	P	RR	LCL	UCL	P	
Stay high vs. stay low									
SO_2	1.42	0.95	2.12	0.09	1.14	0.84	1.55	0.40	0.40
O_3	1.70	1.04	2.77	0.034	1.58	1.09	2.28	0.015	0.81
NO_X	1.07	0.66	1.72	0.80	1.58	1.14	2.20	0.007	0.18
NO_2	1.13	0.73	1.73	0.59	1.49	1.11	2.01	0.008	0.29
CO	0.98	0.62	1.53	0.92	1.31	0.95	1.80	0.10	0.30
$PM_{2.5}$	0.69	0.42	1.13	0.14	1.11	0.78	1.57	0.57	0.13
PM_{10}	0.92	0.59	1.44	0.73	1.14	0.83	1.56	0.41	0.45
Stay moderate vs. stay low									
SO_2	1.17	0.88	1.57	0.28	1.02	0.83	1.26	0.85	0.44
O_3	1.13	0.77	1.65	0.54	1.30	0.99	1.72	0.06	0.55
NO_X	0.96	0.70	1.31	0.79	1.00	0.78	1.27	0.99	0.84
NO_2	0.92	0.68	1.25	0.61	0.98	0.78	1.23	0.85	0.77
CO	0.86	0.64	1.16	0.33	0.91	0.72	1.13	0.39	0.80
$PM_{2.5}$	0.78	0.59	1.04	0.09	0.84	0.66	1.08	0.17	0.69
PM_{10}	1.04	0.77	1.39	0.81	0.83	0.67	1.02	0.08	0.22
Increase vs. stay low									
SO_2	1.26	0.97	1.64	0.08	1.37	1.12	1.67	0.002	0.63
O_3	1.34	0.90	2.01	0.15	2.05	1.51	2.80	<.0001	0.10
NO_X	0.90	0.67	1.19	0.45	1.27	1.01	1.62	0.045	0.06
NO_2	0.93	0.69	1.27	0.66	1.63	1.30	2.04	<.0001	0.004*
CO	1.00	0.73	1.37	0.98	1.57	1.21	2.03	0.001	0.031*
$PM_{2.5}$	0.71	0.55	0.90	0.006	1.03	0.83	1.28	0.79	0.025*
PM_{10}	1.30	1.00	1.69	0.047	1.10	0.91	1.34	0.33	0.32
Decrease vs. stay low									
SO_2	1.19	0.84	1.68	0.32	1.07	0.84	1.38	0.59	0.63
O_3	1.13	0.76	1.67	0.54	1.35	1.02	1.79	0.037	0.47
NO_X	1.05	0.74	1.49	0.79	1.22	0.94	1.57	0.13	0.50
NO_2	1.02	0.73	1.41	0.91	1.21	0.96	1.53	0.10	0.39
CO	0.89	0.64	1.22	0.46	1.02	0.80	1.28	0.90	0.50
$PM_{2.5}$	0.72	0.50	1.03	0.07	1.06	0.80	1.41	0.66	0.09
PM_{10}	0.92	0.63	1.33	0.66	0.99	0.77	1.28	0.96	0.73

* Significance of $p < .05$

Covariates: Preterm birth in first pregnancy; interpregnancy interval; maternal age; race/ethnicity; pre-pregnancy BMI; smoking; alcohol use; parity; insurance status; marital status; asthma history
